# Establishment strategies for poplars, including mulch and plant types, on agricultural land in Sweden

**DOI:** 10.1007/s11056-018-9652-6

**Published:** 2018-06-09

**Authors:** Karin Hjelm, Rebecka Mc Carthy, Lars Rytter

**Affiliations:** 1 The Forestry Research Institute of Sweden (Skogforsk), Ekebo 2250, 268 90 Svalöv, Sweden; 2 NEPCon Certification Aps, Søren Frichs Vej 38K, 8230 Åbyhøj, Denmark

**Keywords:** Browsing, Competing vegetation, Cuttings, Growth, Mulching, Vole damage

## Abstract

Biomass from forestry is one of the largest components of Sweden’s renewable resources. Poplars are currently the highest producing tree species available and are therefore natural choices for biomass-oriented production. Growing poplars has been of most interest on agricultural land, but the knowledge and experience about their cultivation is still limited. Factors that have a large impact on the regeneration results are plant material, competing vegetation, browsing and damage caused by voles or climatic factors. Due to large establishment costs, there is a need to find methods to secure the establishment both biologically and economically. In this study the effect of plastic mulch in combination with three different plant types (short cuttings, long cuttings and rooted plants) were tested at three different sites. Five years after planting, the overall effect of mulch was an improved plant survival and growth. In most cases, long cuttings outperformed short cuttings and rooted plants. Clonal differences were present, indicating the importance of using plant material adapted to site conditions. All sites were heavily affected by browsing and during the experimental period 100% of the plants were damaged at some point. Planting poplars without fencing is therefore doubtful. Results from this study conclude that poplars can be established with success on agricultural land if proper measures are used depending on the site to be planted.

## Introduction

The Nordic countries have announced the goal to develop carbon neutral energy systems by 2050, where woody biomass from the forest sector will be a major source of renewable energy and products in the future society (IEA 2013). There are large areas used for forestry in Sweden and other neighbour countries. Almost 58% of the land area is classified as productive forest land in Sweden (Swedish University of Agricultural Sciences 2017). During the last century, agricultural land has been converted to forest land in the country, but there are still large areas, 300,000–500,000 ha, of abandoned agricultural land that can be used for producing biomass (Larsson et al. 2009). Since the use of woody biomass is expected to increase, an important task is to achieve a sustainable production. Available possibilities were presented in a recent review (Rytter et al. 2016). One suggested option was to introduce and use new tree species. The area-based productivity could be enhanced significantly by changing to fast-growing tree species on forest land and for afforestation of abandoned agricultural land.

The tree species with the highest production under Nordic boreal conditions, seen so far, are found within the genus *Populus*. Poplars have been introduced in Sweden and shown production figures of 7–10 tonnes of stem dry matter ha^−1^ year^−1^ on suitable sites (e.g. Karačić et al. 2003; Christersson 2010; Johansson and Karačić 2011; Tullus et al. 2013), and the selection of fast-growing and climatically adapted plant material (clones) of poplars is proceeding (Stener and Westin 2017). Most poplar plantations are found on converted agricultural land, where the success rate is higher compared to forest land (Engerup 2011). However, problems at the establishment phase are still present. Expensive plants and the lack of safe establishment methods have been recognised as an obstacle for large scale introduction of fast-growing deciduous tree species (Hannerz and Bohlin 2012), so there is a need to continue to develop effective establishment procedures on this type of land.

Poplars need fertile and well-drained soils with continuous water supply to utilize their full growth capacity (Boysen and Strobl 1991; Stanturf et al. 2001; Rytter et al. 2011). The most suitable soils are deep (> 1 m to ground water) and have a medium texture. Soil acidity is critical for unlimited growth. Studies have shown that pH levels below 5 often lead to reduced growth (e.g. Böhlenius et al. 2016; Hjelm and Rytter 2016). As a result of these demands, abandoned agricultural land, with a pH generally above 5, is often used for poplar plantation.

Although often planted with good results, there are some upcoming situations, which may destroy cultivations on agricultural land. The presence of competing vegetation is one factor that negatively affects survival and growth (Albertsson et al. 2014; Löf et al. 2015). Weed control is therefore necessary and a failure to control weeds may also increase the risk for vole damage (DesRochers and Sigouin 2014), a serious threat to establishment success. Species within the *Populus* genera are generally attractive for browsing by ungulates (Månsson et al. 2007; Bergqvist et al. 2014). This problem could be handled by fencing the plantation area at a substantial cost, by hunting wildlife or by planting large areas and thereby reducing browsing intensity (e.g. Bergqvist et al. 2014; Jönsson 2015).

In addition, information on what plant type that best combines a secure establishment with a good economy is still limited. For example: when using cuttings, large cuttings are more expensive than small ones, but it has been shown that increased length (Kaczmarek et al. 2014; Schuler and McCarthy 2015) and thickness of cuttings (Dickmann et al. 1980; Thomas et al. 2016) are positively related to plant development.

The aim of this study was to test mulching as a weed control tool and combine this with different plant types, i.e. cuttings of different lengths and rooted plants, to find the best way to establish poplar on agricultural land. The experimental plots were established in larger commercial plantations to avoid high browsing pressure. Our hypotheses were (a) mulch will reduce the negative effect of weed competition, (b) when using mulch, smaller plant types, i.e. short cuttings, are comparable to larger plant material and (c) fencing is not needed to reduce browsing if large areas are planted.

## Materials and methods

### Study sites and preparation

The experiment was established on three agricultural fields in a west–east direction in southern Sweden (Table 1). The westernmost site was Haneström (58°07′N; 12°09′E; altitude 50 m), where the field was treated with the herbicide glyphosate (Roundup, Monsanto Crop Sciences) in summer 2011. Thereafter, the field was ploughed down to a depth of 30 cm in one pass followed by a harrow to even out the ground and then again treated with herbicides. Since the weeds had returned before planting in June 2012, the plots were harrowed once more. On the central site Åryd (56°51′N; 14°59′Ö; altitude 205 m) all soil preparation measures were performed in spring 2012. The field was first treated with herbicides, then ploughed and harrowed before planting in June. At Dal (58°09′N; 16°45′E; altitude 15 m), the eastern site, the area was treated with glyphosate in summer 2011 prior to ploughing. In the autumn, the field was again treated with the herbicide. In spring 2012 the site was harrowed before planting in May.

**Table 1:** Information on the study sites

Site	Stand (ha)	Area (ha)	Soil texture	pH (H_2_O)	pH (CaCl_2_)	Prec. (mm)	Temp. (°C)	Veg. length (days)
Haneström (western)	4.9	1.4	Glacial fine clay	6.2	5.1	800–900	6–7	190–200
Åryd (central)	3.0	1.4	Sandy moraine	5.8	5.0	600–700	6–7	190–200
Dal (eastern)	21.8	2.1	(Post)glacial clay	6.3	5.6	500–600	6	190–200

Size of the total stand area (Stand, ha) and sized of the experimental area (Area, ha). Soil texture was estimated from a pooled sample (two samples per block) collected from the soil layer 0–20 cm. Soil pH was recorded with two methods: distilled H_2_O and CaCl_2_-solution. The data on mean annual precipitation (Prec.), annual mean temperature (Temp.), and annual vegetation length (Veg.) are given as averages for the period 1961–1990 from the Swedish Meteorological and Hydrological Institute (SMHI)

Before soil preparation, two soil samples were collected in each block down to 20 cm depth. The samples were pooled block-wise, soil texture was estimated by rolling test and soil pH was analysed in distilled H_2_O and 0.01 M CaCl_2_ solution (Table 1).

### Experimental design and plant material

**Fig. 1:**
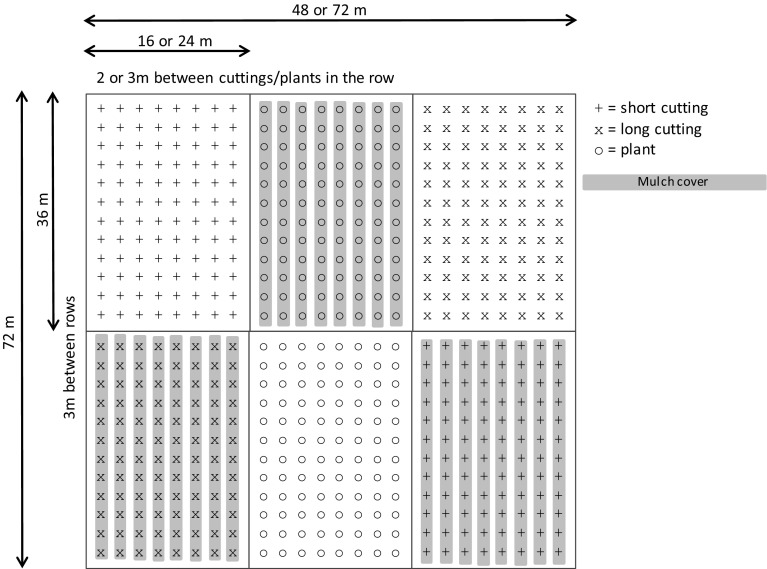
The research layout in a block with 3 plots with plastic mulch and 3 plots with no mulch. Each plot contained 96 plants of one of each plant type; short cutting, long cutting or rooted plant

Four blocks were placed embedded in commercial poplar plantings at each site. A block was divided into six plots where a combination of two mulch treatments (mulch and no mulch) and three seedling types were applied (Fig. 1). In the treatment with mulch, the soil got slightly elevated in rows by a tractor having a developed “potato plough” that applied brown herbistatic polyethylene mulch (thickness 45 µm, width 90 cm, hereafter called mulch) in complete strings on three of the six plots. Approximately half of the mulch was covered by soil when it was applied with the plough. On the other three plots bare soil was left after ploughing (no mulch). On each mulch treatment plot (mulch or no mulch), one of three plant types were planted: (1) short poplar cuttings (18 cm), (2) long poplar cuttings (50 cm) and (3) containerized plants from rooted poplar cuttings (mean height 44 cm, hereafter called plants). Each plant type consisted of twelve commercially available clones (Table 2), that were randomly planted within eight rows (in total 96 plants per plot). For the rooted plants, the mean plant height among the clones varied from 27 to 55 cm. In total, 3 sites × 4 blocks × 2 mulch treatments × 3 plant types × 96 plants = 6912 plants were planted in the experiment.

**Table 2:** Information on the clones used in the study

Clone	Skogforsk ID	Taxonomy^a^	Additional information
1	S21K766049	*P. trichocarpa*	
3	S21K766003	*P. maximowiczii* × *P. nigra*	Commercial name Rochester
4	S21K82604	*P. trichocarpa*	
5	S21K766048	*P. trichocarpa*	
6	S23K9040086	*P. maximowiczii* × *P. trichocarpa*	
7	S23K9040089	*P. maximowiczii* × *P. nigra*	
8	S23K9040073	*P. trichocarpa*	
9	S23K9040025	*P. trichocarpa*	
10	S23K9040019	*P. trichocarpa*	
11	S23K9040011	*P. trichocarpa*	
12	S23K9040006	*P. trichocarpa*	
14	S216PPL52	*P. trichocarpa*	

All clones are commercially available in Sweden. Clone is the identification number used in this study and Skogforsk ID is the identification used by the Forestry Research Institute of Sweden (Skogforsk)

^a^*P. maximowiczii* (Henry), *P. nigra* (Linneaus), *P. trichocarpa* (Torrey and Gray)

All cuttings originated from 1-year-old shoots. The rooted plants had been growing in containers in a nursery for one growing season before planting, while the cuttings were cut in the winter and directly put in storage. All plant material was stored in freezer (− 2 to − 4 °C) during winter and moved to a refrigerator in May (4 °C for about 2 weeks). The cuttings were placed in water during 1–2 days before planting. The short cuttings were planted by hand with only one bud located above the soil surface. For the long cuttings, a preformed planting hole was made with a stick and the cuttings were planted with approximately one-third of the cutting length (about 15 cm) above soil surface. The rooted plants were planted with a conventional planting tool (Pottiputki). After planting in the middle of the mulch strings in the plastic mulch, the perforated holes were covered by soil to minimize moisture losses.

### Measurements

Measurements were performed during late winter the first two years and in autumn of year 3. On each occasion, heights of living plants (cm), from soil surface to the base of the highest located bud, were recorded. In addition, survival and degree of damage (in four classes: 1 = light damage, 2 = damaged, 3 = severely damaged, and 4 = dead or dying) and cause of damage (e.g. browsing, rubbing, etc.) were assessed.

**Fig. 2:**
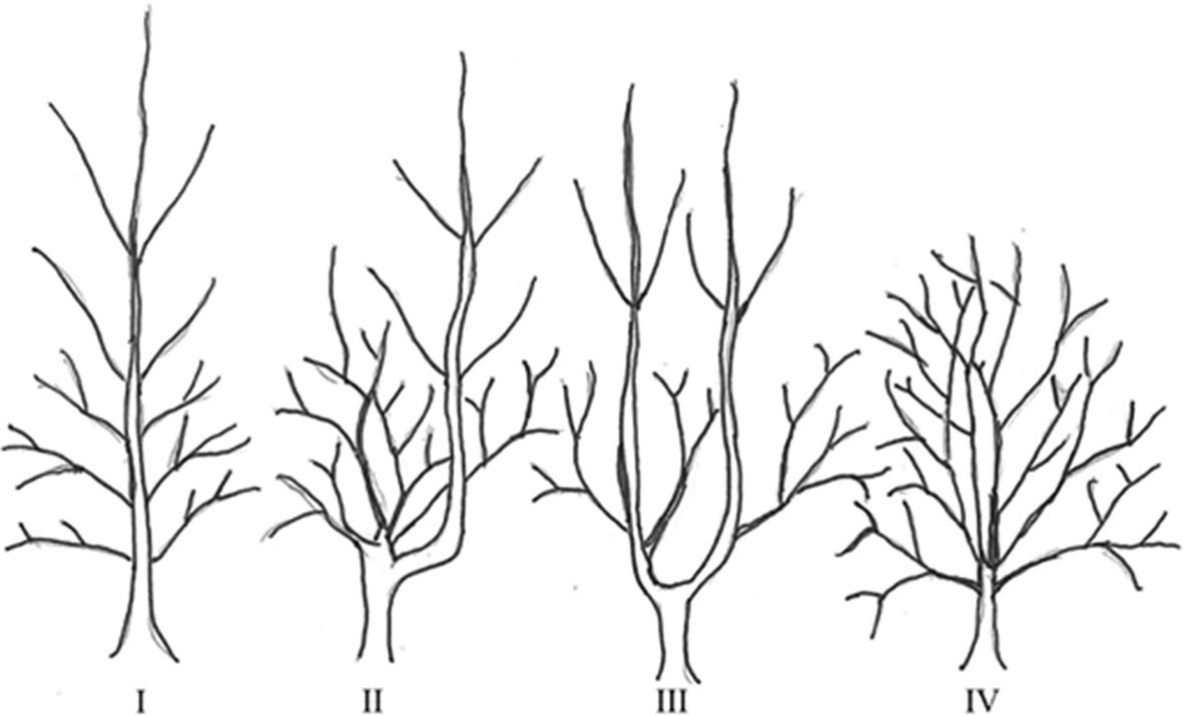
Illustration of the stem forms divided into four classes: (I) straight, (II) crooked, (III) multiple tops/shoots (≤ three), and (IV) bushy (≥ four tops/shoots)

The last measurement was done in winter 2017 when the plants had grown for five years. Tree height (cm), survival, vitality and stem form were recorded. Vitality was assessed in three classes: (a) vital, (b) reduced vitality, and (c) dying. Stem form was assessed in four classes (Fig. 2): (I) straight, (II) crooked, (III) multiple leading tops/shoots (≤ three), and (IV) “bushy” (≥ four leading tops/shoots or lack of leading tops/shoots).

### Statistical analyses

Different statistical models were applied for tree responses depending on the distribution of the input data and purpose of the analyses. When analysing effects of the explanatory variables on the proportion (η) of survival after five growing seasons, the input data followed a binomial distribution so generalized linear mixed models implemented in PROC GLIMMIX in SAS 9.4 (SAS Institute, Cary, NC, USA) were applied:

1 $${\text{logit(}}\upeta_{\text{ijkl}} )= \log \left( {{{\upeta_{\text{ijkl}} } \mathord{\left/ {\vphantom {{\upeta_{\text{ijkl}} } {(1 -\upeta_{\text{ijkl}} )}}} \right. \kern-0pt} {(1 -\upeta_{\text{ijkl}} )}}} \right) =\upmu +\upalpha_{i} + {\text{b}}_{\text{ij}} +\upgamma_{\text{k}} +\upalpha_{\text{i}}\upgamma_{\text{k}} +\updelta_{\text{l}} +\upalpha_{\text{i}}\updelta_{\text{l}} +\upgamma_{\text{k}}\updelta_{\text{l}} +\upalpha_{\text{i}}\upgamma_{\text{k}}\updelta_{\text{l}} +\upvarepsilon_{\text{ijkl}}$$

where µ is the general mean, α_i_ is the fixed effect of site (*i* = 1–3), b_ij_ is the random effect of block (*j* = 1–4) within site, γ_k_ is the fixed effect of mulch treatment (*k* = 1–2) and δ_l_ is the fixed effect of plant type (*l* = 1–3). The interactions between site, mulch treatment and plant type were included in the model as fixed effects. Damage by deer/moose was also analysed using the model described above. At Haneström, vole damage reached high levels and was therefore analysed to detect possible differences among treatments (but in this specific case site was removed from the model).

All living trees were used when analysing tree height (cm). The height followed a normal distribution with equal variances and was analysed with a mixed model implemented in PROC MIXED in SAS 9.4:

2 $${\text{Y}}_{\text{ijkl}} =\upmu +\upalpha_{\text{i}} + {\text{b}}_{\text{ij}} +\upgamma_{\text{k}} +\upalpha_{\text{i}}\upgamma_{\text{k}} +\updelta_{\text{l}} +\upalpha_{\text{i}}\updelta_{\text{l}} +\upgamma_{\text{k}}\updelta_{\text{l}} +\upalpha_{\text{i}}\upgamma_{\text{k}}\updelta_{\text{l}} +\upvarepsilon_{\text{ijkl}}$$

where the explanatory variables were the same as in Eq. 1. Satterthwaite approximation was used to determine the appropriate degrees of freedom in all analyses. When significant differences between treatment means were detected, they were separated using least square means with *p* values adjusted according to Tukey–Kramer. An α-level of 0.05 was used in all analyses.

Clonal effects for height and survival were also analysed in this experiment. In this case, clone was added as a fixed effect in the models above and included in interactions with site, mulch treatment and plant type. For survival, the model with clones did not converge due to imbalances and therefore site was excluded as fixed effect and only used in the random block within site effect.

The outcomes of cause of damage and stem form classes were described using frequency distribution per site, mulch treatment and plant type. For this, the FREQ procedure in SAS 9.4 was used.

## Results

### Survival and damage

**Fig. 3:**
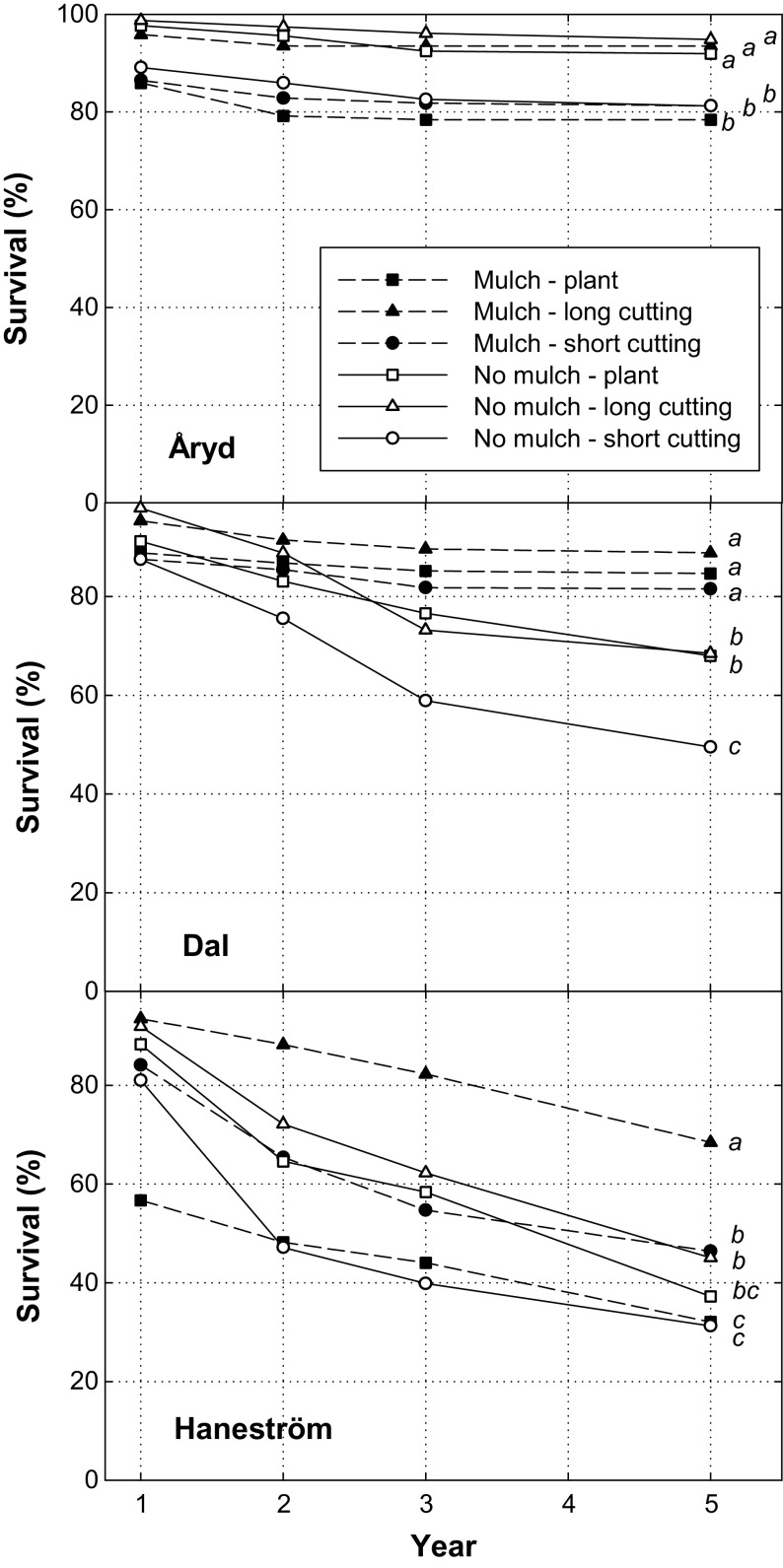
Plant survival during the experimental period. At each site mulch and no mulch were used in combination with three plant types: short cutting (18 cm), long cutting (50 cm) and rooted plant. The treatment combinations shown in the figure are mulch and plant (dashed line and filled square), mulch and long cutting (dashed line and filled triangle), mulch and short cutting (dashed line and filled circle), no mulch and plant (solid line and open square), no mulch and long cutting (solid line and open triangle) and no mulch and short cutting (solid line and open circle). Different letters show significant differences between treatment combinations at the inventory year 5

Plastic mulch increased poplar survival by 10%, from 63% without to 73% with mulch five years after planting. There was also a significant plant type effect, where the long cuttings had a higher survival (76%), compared with rooted plants (65%) and short cuttings (62%). Even though there were significant main effects of both mulch and plant type, the effects on survival differed depending on site (Fig. 3, Table 3). At Åryd, the overall survival was rather high five years after planting and no treatment combination was below 78%. Most of the mortality occurred during the first season after planting and thereafter only few plants died. The greatest survival was found for long cuttings, irrespective of mulch treatment, with over 90% survival, while short cuttings showed about 10% lower survival (Fig. 3). The variation in survival between treatment combinations was larger at Dal and Haneström. At Dal, mulch had a positive effect on survival, and long cuttings and rooted plants had an advantage compared with short cuttings. At Haneström, plant mortality was high, and only long cuttings planted in mulch achieved a survival above 50% (68%).

**Table 3:** Results of analyses of survival and height five years after planting

Effect	*p* value	
	Survival	Height
Site	0.0001	0.0001
Mulch	0.0001	0.0001
Site × mulch	0.0001	0.0001
Plant	0.0001	0.0001
Site × plant	0.0001	0.0009
Mulch × plant	0.0001	0.1419
Site × mulch × plant	0.0826	0.2733

The main reason behind the sometimes low survival was the high frequency of damaged plants within the experiment. Almost all planted plants were damaged to some degree during the first two years after planting. Fewer plants were damaged at Åryd during the third growing season, where the frequency varied between 16 and 28% for the treatment combinations. This decline in damage was not found for Dal and Haneström, where almost all seedlings received some damage even during the third growing season. During the experimental period, almost all plants had received some kind of damage at least once, resulting in nearly 100% damaged plants in total. Therefore, it was not possible to find any treatment effect of either site, mulch treatment or plant type on damage.

**Fig. 4:**
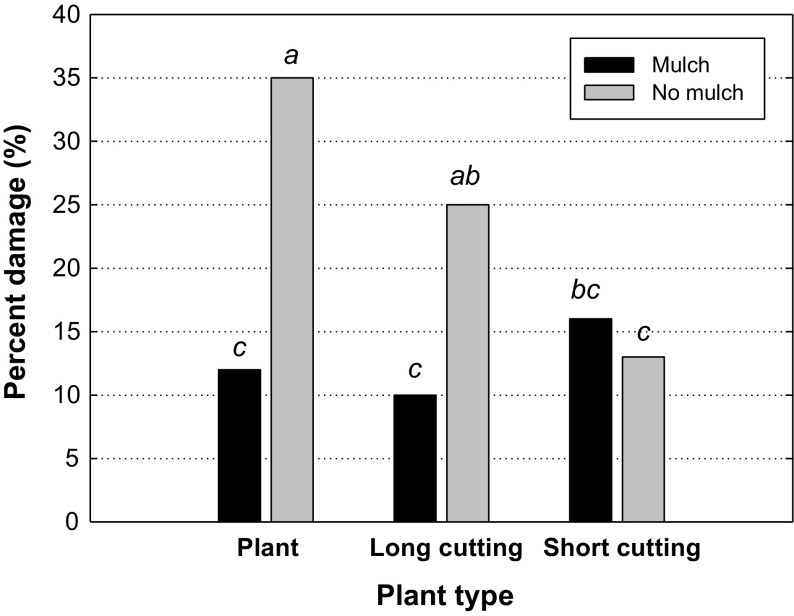
Vole damage (%) at Haneström for the combination of mulch treatment (mulch is shown as black columns and no mulch as grey columns) and plant type. Different letters indicate significant differences between treatment combinations

The cause of damage was to a large extent browsing by deer or moose (Table 4). During the first year in Åryd, browsing by hare was also frequent, while frost was common at Dal during the first two years. At Haneström, damage by frost and vole caused problems. Almost all plants had been browsed by deer or moose more than once during the experimental period, but the analyses showed a significant effect of plant type (*p* = 0.0017), where 85% of the short cuttings were browsed, which was lower compared with long cuttings and plants, both 90%. A significant site × mulch interaction (*p* = 0.0001) showed that at Åryd, plastic mulch reduced damage by deer and moose from 90% without mulch to 80% with mulch. At the two other sites mulch had no significant effect on browsing. Voles were an important damage agent at Haneström and treatment effects on damage by voles were therefore compared at this site. Here, plastic mulch had a reducing effect on damage, at least in combination with rooted plants and long cuttings (*p* = 0.0001) (Fig. 4).

**Table 4:** Frequency (%) of cause of damage per site and year

Cause	Åryd			Dal			Haneström		
	Year 1	Year 2	Year 3	Year 1	Year 2	Year 3	Year 1	Year 2	Year 3
Deer/moose	58	82	67	64	53	93	45	33	95
Hare	23	0	0	1	0	0	14	6	0
Vole	0	0	0	0	13	0	0	31	0
Frost	8	1	0	24	17	1	34	10	0
Vegetation	0	0	0	0	4	0	0	11	0
Unknown	11	14	33	11	13	6	12	9	5

### Height

**Fig. 5:**
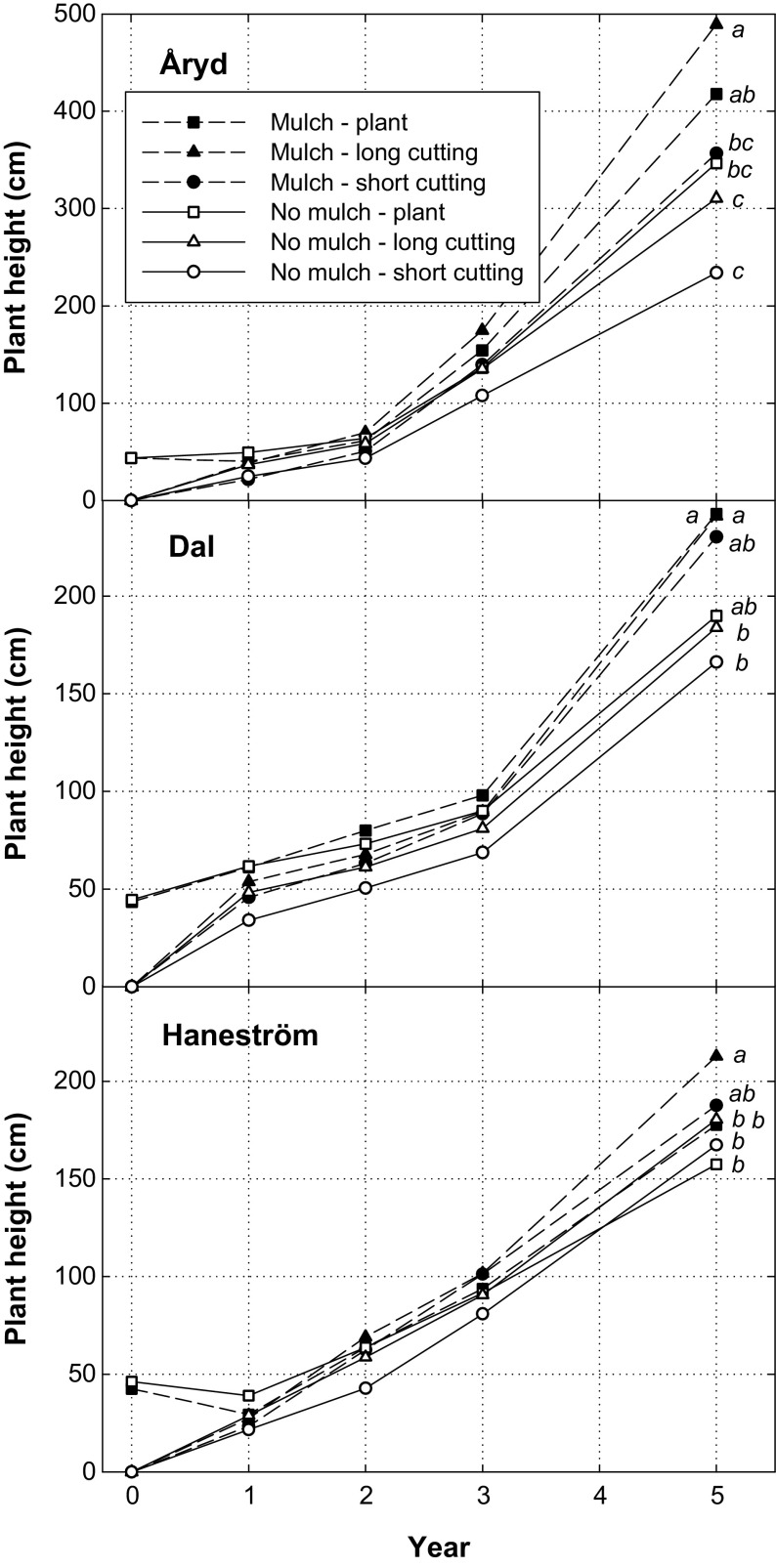
Plant height development at the three sites during the experimental period. At each site mulch and no mulch were used in combination with three plant types: short cutting (18 cm), long cutting (50 cm) and rooted plant. The treatment combinations shown in the figure are mulch and plant (dashed line and filled square), mulch and long cutting (dashed line and filled triangle), mulch and short cutting (dashed line and filled circle), no mulch and plant (solid line and open square), no mulch and long cutting (solid line and open triangle) and no mulch and short cutting (solid line and open circle). Different letters show significant differences between treatment combinations at the inventory year 5. Note that the height scale of Åryd is different from Dal and Haneström

Tree height five years after planting was affected both by site, mulch, plant type and their interactions (Table 3). The trees were highest at Åryd, on average 359 cm, which was double the height measured at the same time at Haneström (181 cm). At Dal the mean height was 209 cm. Plastic mulch had an overall positive effect on tree height, 284 cm with mulch compared with 215 cm for no mulch. Long cuttings were tallest (270 cm), compared with rooted plants (255 cm) and short cuttings (224 cm). However, the response in height differed depending on site (Fig. 5), and there were greater treatment effects at Åryd than at Dal and Haneström. Height development was low at all sites during the first years after planting. After two years the trees were still below 1 m height. The main reason to poor height development was browsing damage. During the third growing season, height increased more rapidly at Åryd, while it was still rather low at Dal and Haneström. This was partly due to the lower damage frequency at Åryd year three, which gave the trees a possibility to recover.

### Stem form

Due to the high browsing pressure, many trees achieved a stem form with several stems (class 3) or a bushy form where no main stems were visible (class 4) (Table 5). This was especially seen at Haneström, where only 22% of the trees were classified with one single main stem and 35% were formed like bushes after five years. Overall, the distribution of stem form classes where rather similar among mulch treatments and plant types.

**Table 5:** Percentage of trees within each stem form class five years after planting

Treatment	Stem form class (%)			
	1-straight	2-crooked	3-multiple	4-bushy
Åryd	37	11	42	10
Dal	53	10	32	5
Haneström	22	18	25	35
Mulch	38	11	35	16
No mulch	42	13	35	10
Plant	37	15	36	12
Long	38	12	36	14
Short	46	8	32	14

Values are shown for each site, mulch treatment and plant type

### Clone effects

**Fig. 6:**
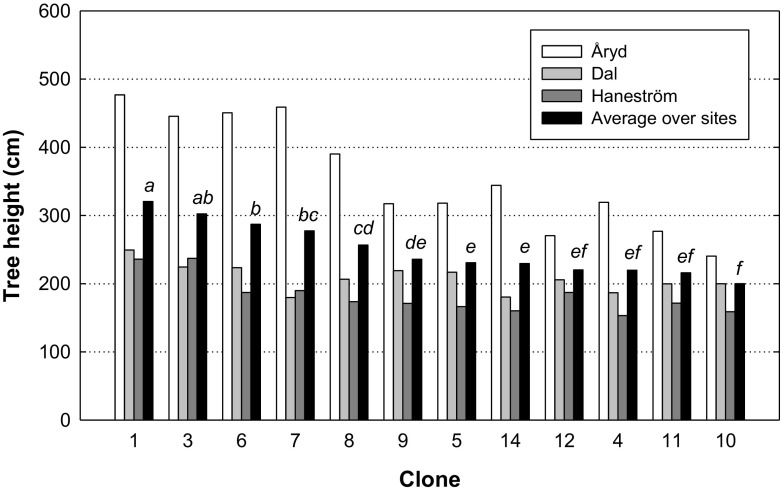
Mean height (cm) of clones five years after planting at the three sites (Åryd is shown as a white column, Dal as a light grey column and Haneström as a grey column) and as the average over all sites (black column). Different letters show differences between average clone height over all sites

A significant effect of clones on height after five years and a significant interaction effect for site and clone (*p* = 0.0001 for both) were found. Clonal effects were stronger at Åryd compared with Dal and Haneström, and the best clones were more than twice the size of the worst performing ones (Fig. 6).

**Fig. 7:**
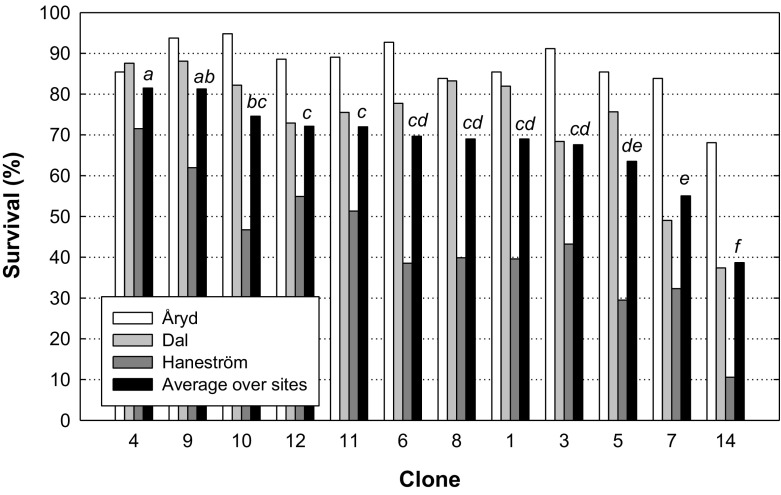
Survival (%) of clones five years after planting at the three sites (Åryd is shown as a white column, Dal as a light grey column and Haneström as a grey column) and as the average over all sites (black column). Different letters show differences between average clone survival over all sites

A significant clone effect was also found for survival (Fig. 7). Although site effects were not possible to analyze statistically due to few replicates, survival was high for most clones at Åryd, while at Haneström the variation in survival among clones was large. Significant interactions with mulch and plant types occurred, and these effects showed that mulch increased survival and decreased individual differences between clones. Regarding plant type, long cuttings had the highest survival irrespective of the clone used. The range between short cuttings and rooted plants changed somewhat depending on the clone used, but the differences were not greater than a few percent and thus of less importance.

## Discussion

### Mulching and soil conditions

This study showed that mulch generally had a positive effect on both survival and growth of poplar on agricultural land (Figs. 3, 5). In addition to reduced competition, it was also seen that mulching reduced browsing and gnawing damage in some cases. Establishment of trees, including poplars, is greatly improved by reducing the negative influence of competing vegetation (e.g. Hansen et al. 1983; Davies 1987; Bilodeau-Gauthier et al. 2011; Böhlenius and Övergaard 2015a) and the positive effect of weed control gets higher with increased site fertility (Pinno and Bélanger 2009). Earlier studies on the use of mulching have shown both positive and negative effects. The varying results partly depend on the mulch itself, since different materials can be used and executed differently. Sometimes sheets of different materials are used (Siipilehto 2001; Hytönen and Jylhä 2013) and sometimes it is applied in long complete strips (Bowersox and Ward 1970; Green et al. 2003; Rytter and Stener 2015), like in this study. Siipilehto (2001) found positive effects on growth of aspen seedlings with sheet mulch, but also negative effects were seen since voles nested below the mulch, destroyed it and damaged plants. Davies (1988) reported, for ash and hornbeam, that opaque mulches were preferable to translucent ones and that the ability to resist weed invasion was more important than enhanced temperature below the mulch. Thus, tree growth was better with impermeable than permeable translucent mulches, probably as an effect of retained moisture. A positive effect of mulch compared with herbicides in survival and shoot growth of hybrid poplar was reported by Bowersox and Ward (1970), who assigned this to better moisture conditions below the mulch during periods of average rainfall or limited drought. However, during prolonged drought mulching prevented remoistening of the soil. In our study we observed comparably low initial survival with mulch, most clearly seen for plants at Haneström (Fig. 3). This was most probably due to dry soil conditions at the time of mulch application and these conditions were preserved by the mulch. Young plants with a rapid demand of soil water are sensitive to dry soil conditions.

Green et al. (2003), who considered both biological and economic aspects, concluded that mulching could improve early growth in short rotation forestry and be advantageous on less fertile sites, but could not recommend it on high-quality sites due to rapid attrition of mulching benefits and a high need for additional weed control. The most important feature of sheet mulches is their ability to decrease plant mortality according to Hytönen and Jylhä (2013). They used small sheets in a trial with silver birch. With larger protection areas, i.e. larger sheets, also the growth of trees can be enhanced by providing favourable soil moisture and temperature conditions (Davies 1987; Vares et al. 2003). In addition, mulching has been shown more durable in controlling weeds than herbicides (Siipilehto 2001) or manual methods (Böhlenius and Övergaard 2015a). Results from the present study in combination with earlier findings show that competing vegetation is one of the key factors when it comes to establishing poplars. It is known from practice in Sweden that repeated weed control could work well, but the advantage with mulching is that it is probably not necessary to come back for control measures and could thereby offer a good economic and sustainable alternative.

The sites used in this study were all probably well suited for afforestation with poplar, i.e. fertile and well-drained soils with continuous water supply (Boysen and Strobl 1991, Stanturf et al. 2001). They were all former agricultural land that had recently been in use for agriculture crops. The pH, which should preferably be above 5 (Ericsson and Lindsjö 1981; Böhlenius et al. 2016; Hjelm and Rytter 2016), was sufficiently high. Thus, there was no reason to believe that soil properties, at least regarding acidity and water availability, should have had a negative influence on the results of this study. In addition, the nutrient availability is usually high on abandoned agricultural land, although nutrient conditions were not analysed in this study. Instead, the superior growth at Åryd was probably explained by reduced browsing damage from year three and onward, which gave the trees a possibility to recover.

The conclusion is that soil preparation, mulch material, performance and site conditions must be carefully combined to achieve best results for establishing plants (cf. Green et al. 2003). Mulch generally worked well, but the soil moisture conditions at application of mulch needs attention. A commercial concept was used in this study, which worked satisfactorily but could be improved. It is important to select a mulch material that can protect the soil long enough but thereafter is fully biodegradable. In this study, the mulch used was partly degraded but still visible after five years.

### Plant types

Among the tested plant types, short cuttings were generally inferior to long cuttings and rooted plants (Figs. 3, 5). This is in agreement with what is usually found in the literature. Rossi (1991) reported a clear trend saying that longer cuttings of hybrid poplar survived and grew better than short cuttings. The study was performed with 10–50 cm long cuttings on agricultural land. Phillips et al. (2014) found that 3 m poles produced more biomass and had the best root spread and root length compared to 1 m wands and 0.5 m stakes of poplar and willow. Mc Carthy et al. (2017) found that rooted plants and long cuttings (50 cm) of poplar performed better than short cuttings (20 cm) on forest land, and especially in combination with mounding. In the present study, the same plant material was used, but on agricultural land that is different from forest land both regarding soil characteristics and the methods that could be used for site preparation. The results regarding the plant material are similar in both studies, which support the findings that larger plant material is to prefer in various environments. On the contrary, in the studies by Böhlenius and Övergaard (2015b, 2016) cuttings had better or equal establishment and early growth compared with rooted plants on agricultural land but not on forest land. Forest soils are more heterogenous and constraining factors are not possible to control in the same way as on agricultural land, and this may be a reason to why already rooted plants seems more suitable. Longer poplar cuttings may also be at risk at establishment in drier climate since a large leaf mass will increase transpiration and thereby lead to water stress (Vigl and Rewald 2014). However, this is much a question of planting depth and the relation between biomass above and below ground. If planted deep enough to reach moist soil layers, longer cuttings might be better in a drier climate, which has been found in Mediterranean climate (Chirino et al. 2009).

In addition to cutting length also the cutting diameter influences poplar performance. Dickmann et al. (1980) showed significant positive effects on poplar survival and growth when the cutting diameter was increased and suggested that cuttings used for planting should be over 6 mm in diameter. Temperature is an abiotic factor that may affect the use of cuttings since low soil temperatures reduces the rooting ability of poplars (Landhäusser 2003; Zalesny et al. 2005). Rooted plants could therefore be the best choice on cold sites where the initiation of roots may be hampered by low temperature.

In conclusion and based on the varying results from the literature and this study, the best combination in practice for successful establishment of poplar on agricultural land seems to be reasonably large plants/cuttings together with a treatment, such as mulch, that avoids competition from weeds.

### Wildlife damage

A common idea with afforestation is that large planted areas with high forage availability should reduce the browsing pressure and allow a good establishment with limited damage. This effect has been shown in the literature (Bergqvist et al. 2014; Jönsson 2015). However, this finding was not supported in this study, where heavy browsing by deer and moose was observed on all sites even if the total planted area was up to 22 ha (Table 4). A better knowledge of the local wildlife situation is obviously necessary when planning plantations of *Populus* species. We could see little effect of treatment combinations on browsing since almost all plants were attacked, but an interesting result was the lower browsing of ungulates found on short cuttings compared to the other plant types. One could suspect a lower browsing height to be a factor, but later height measurements could not support any effect of less browsing (Fig. 5). Once the plants were established and had developed their root systems they showed an ability to outgrow the browsing animals. The inventory after 5 years clearly showed that height growth had accelerated, and the plants had reached a height that was difficult to reach for deer (Fig. 5). Netzer (1984) also observed the ability for poplar to grow out of reach for deer under high browsing pressure. Another possibility to reduce browsing damage in the establishment phase is to plant whips or large poles that are above browsing height (cf. Stanturf & van Oosten 2014), but this has to be further investigated since rather great heights are needed to reduce at least damage by moose.

Browsing by ungulates is an obstacle for plant development, but vole attacks may be even more severe for plant survival on afforested farmland (Rytter and Lundmark 2014). Damage by voles may be an important economic factor, as exemplified by estimates from Finland (Huitu et al. 2009). Damage can be reduced by removing weeds, as shown with mounding on agricultural land (DesRochers and Sigouin 2014). In this study, problems with vole gnaws were seen at Haneström (Fig. 3), where competition from grass was high.

### Performance of clones

This study revealed differences among the poplar clones in both survival and height (Figs. 6, 7). This could be expected and is important to evaluate when selecting clones for future afforestation and regeneration (Kaczmarek et al. 2013; Stener and Westin 2017). However, we could not see a relation between survival and plant height. Instead, clones with high survival did not perform particularly well when looking at plant height. Our interpretation was that more robust clones regarding survival could continue to grow also as smaller plants, while those were dead within the sensitive clones. This could give a somewhat opposite effect between survival and plant height. In genetic tests, Stener and Westin (2017) found that the poplars used in Scandinavia (*P*. *trichocarpa*, *P*. *balsamifera*, *P*. *maximowiczii* and their hybrids) were inferior to hybrid aspen (*P*. *tremula* × *P*. *tremuloides*), both in survival and initial growth. This stresses the importance of continuing with breeding work of poplar since it has a documented high growth potential (Karačić et al. 2003; Christersson 2010; Johansson and Karačić 2011; Tullus et al. 2013). Blake et al. (1996) concluded that the water situation is critical for poplar survival since there is a small margin of safety from critical levels of cavitation and this varies among poplar species and clones. Future breeding work should take this into account (cf. Monclus et al. 2009; Krabel et al. 2015), as well as rooting ability contrary production.

## Conclusions

This study has shown, in accordance with previous experience (e.g. Stanturf et al. 2001), that absence and removal of weeds is a prerequisite for fast and problem free establishment of poplars. The existence of weeds increases loss of plants and delays their development. It also harbours voles, which may be a severe obstacle for establishment success. Although weeds do not directly affect browsing animals, they increase the time at which browsing may occur due to competition and therefore slower height growth of plants. However, even with low weed competition fencing is frequently needed. This study showed that by using large balanced cuttings or plants of selected clones, in combination with plastic mulch, it was possible to reduce the effect of weeds and shorten the time period where browsing damage is critical.
